# Revisiting the gonadotropic regulation of mammalian spermatogenesis: evolving lessons during the past decade

**DOI:** 10.3389/fendo.2023.1110572

**Published:** 2023-04-14

**Authors:** Indrashis Bhattacharya, Souvik Dey, Arnab Banerjee

**Affiliations:** ^1^ Department of Zoology, School of Biological Science, Central University of Kerala, Kasaragod, Kerala, India; ^2^ Manipal Centre for Biotherapeutics Research, Manipal Academy of Higher Education, Manipal, Karnataka, India; ^3^ Department of Biological Sciences, Birla Institute of Technology and Science (BITS) Pilani, Goa, India

**Keywords:** gonadotropins, blood-testis barrier, male fertility, spermatogenesis, infertility

## Abstract

Spermatogenesis is a multi-step process of male germ cell (Gc) division and differentiation which occurs in the seminiferous tubules of the testes under the regulation of gonadotropins – Follicle Stimulating Hormone (FSH) and Luteinising hormone (LH). It is a highly coordinated event regulated by the surrounding somatic testicular cells such as the Sertoli cells (Sc), Leydig cells (Lc), and Peritubular myoid cells (PTc). FSH targets Sc and supports the expansion and differentiation of pre-meiotic Gc, whereas, LH operates *via* Lc to produce Testosterone (T), the testicular androgen. T acts on all somatic cells e.g.- Lc, PTc and Sc, and promotes the blood-testis barrier (BTB) formation, completion of Gc meiosis, and spermiation. Studies with hypophysectomised or chemically ablated animal models and hypogonadal (hpg) mice supplemented with gonadotropins to genetically manipulated mouse models have revealed the selective and synergistic role(s) of hormones in regulating male fertility. We here have briefly summarized the present concept of hormonal control of spermatogenesis in rodents and primates. We also have highlighted some of the key critical questions yet to be answered in the field of male reproductive health which might have potential implications for infertility and contraceptive research in the future.

## 1 Introduction

An alarming decline in the sperm count of men has become a global concern (1). Spermatogenesis occurs within testicular seminiferous tubules under the regulation of gonadotropins – Follicle Stimulating Hormone (FSH) and Luteinising hormone (LH) and involves regulated division and differentiation of male germ cells (Gc) to sperm (2). In mammals, it is a multi-step event that includes i) establishment of spermatogonial stem cells (SSC) ii) self-renewal and differentiation of SSC to form spermatogonial progenitor cells (SPC) iii) spermatogonial expansion and differentiation, iv) meiotic initiation of differentiated spermatogonia v) meiotic progression of spermatocytes to spermatids vi) maturation of spermatids to spermatozoa and vii) spermiation (3). This entire process is extremely rapid (around 35 days in mice, 52 days in rats, 46 days in rhesus macaque and 64 days in humans) with incredible intrinsic speed (1000 sperm/sec) (3).

The hypothalamo-hypophysial-testicular axis (HHT axis) is a three-tier neuro-endocrine circuit with hierarchical regulatory cascades (both stimulatory and inhibitory feedback loops) (4). Under the influence of hypothalamic KNDy (K= Kisspeptin, N= *Neurokinin B* and Dy = Dynorphin) neurons, specific nuclei located at mediobasal/preoptic/arcuate/infundibular area synthesize and release decapeptide GnRH in a pulsatile manner (5). The GnRH further stimulates pituitary-gonadotrophs to secrete gonadotropins (LH and FSH). The differential pulse frequency and amplitude of GnRH, selectively augments either LH or FSH (high and low frequencies favor LH and FSH respectively) release (5). LH acts on the interstitial Leydig cells (Lc) to produce the testicular androgen—testosterone (T) (6). Sertoli cells (Sc) are the major component of the seminiferous tubules that express the receptors for both FSH (FSH receptor, FSH-R) as well as T (androgen receptor, AR) and provide critical micro-environment for Gc nourishment and differentiation (6). Sc-produced inhibin and Lc-generated T selectively suppress the release of FSH from the pituitary and GnRH from the hypothalamus respectively (4–6).

Within twenty years of their identification (7), clinical cases of familial hypogonadism due to isolated gonadotropic deficiency started to get reported frequently (8, 9). In 1971, GnRH (previously known as LHRH) was purified and subsequently got recognized for the Nobel Prize in 1977 (10–12). The same year, a naturally occurring mutation in GnRH [termed as hypogonadal (*hpg*)] was reported in mice confirming the absolute necessity of gonadotropins in gonadal functions and gametogenesis (13). During the 1980s to mid-1990s classical endocrinological studies employed hypophysectomised or GnRH-depleted (either immunologically or pharmacologically) animal models supplemented with purified or recombinant gonadotropins (either alone or in combination) indicating the probable functions of FSH and LH (via T) in spermatogenesis (14–17). From the late 1990s, the success of genetically manipulated mouse models (both gain-in-function or knockout strategies) has further revealed the selective and synergistic role(s) of FSH and LH in regulating male fertility (18–21). This article briefly discusses the critical gonadotropic control of spermatogenesis. We further highlight currently unanswered areas in gonadotropin biology having potential implications on male infertility and contraceptive research.

**Figure 1:**
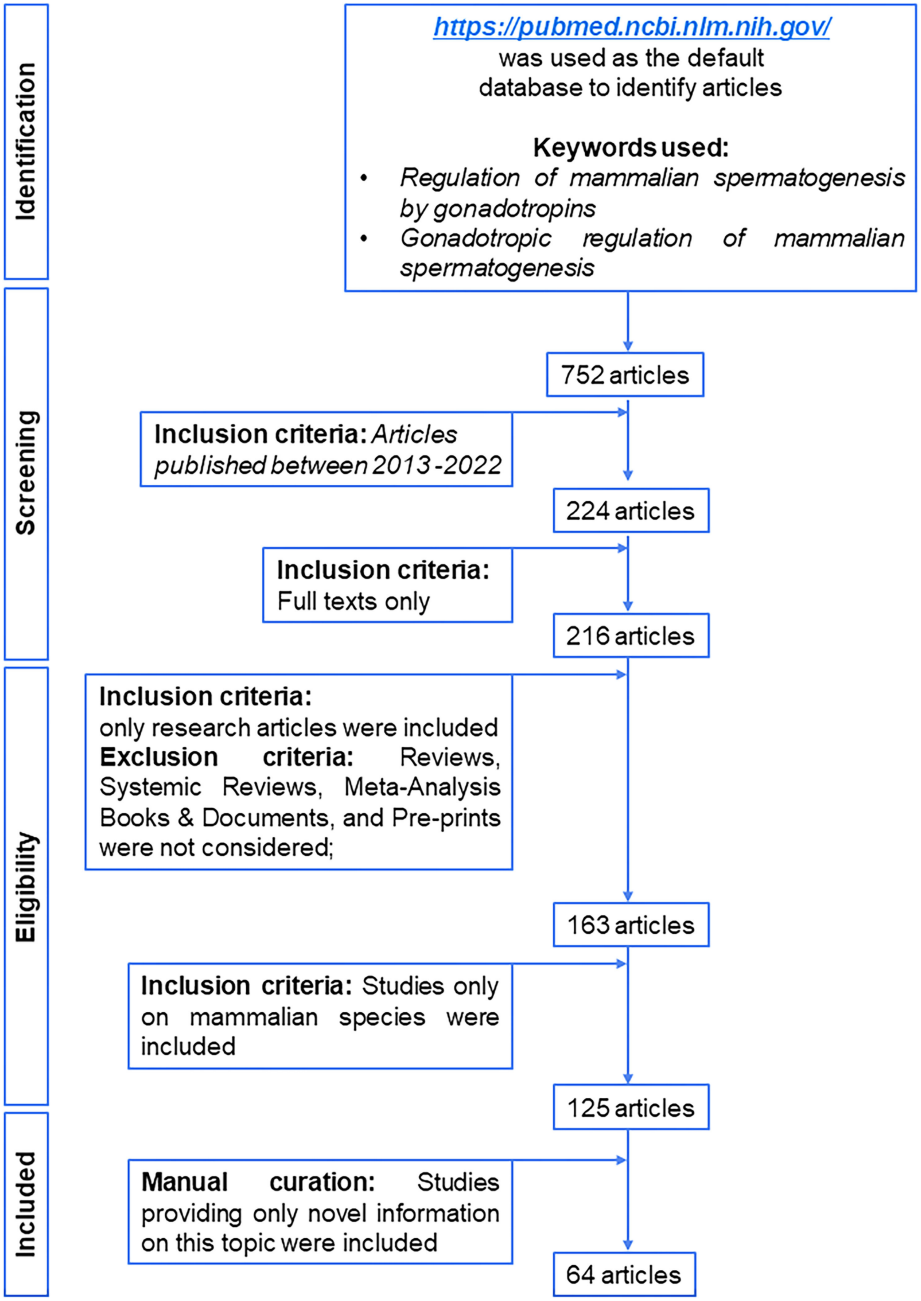
PRISMA flow diagram of selection of articles published in last decade related to gonadotropic regulation of spermatogenesis in mammals.

**Figure 2:**
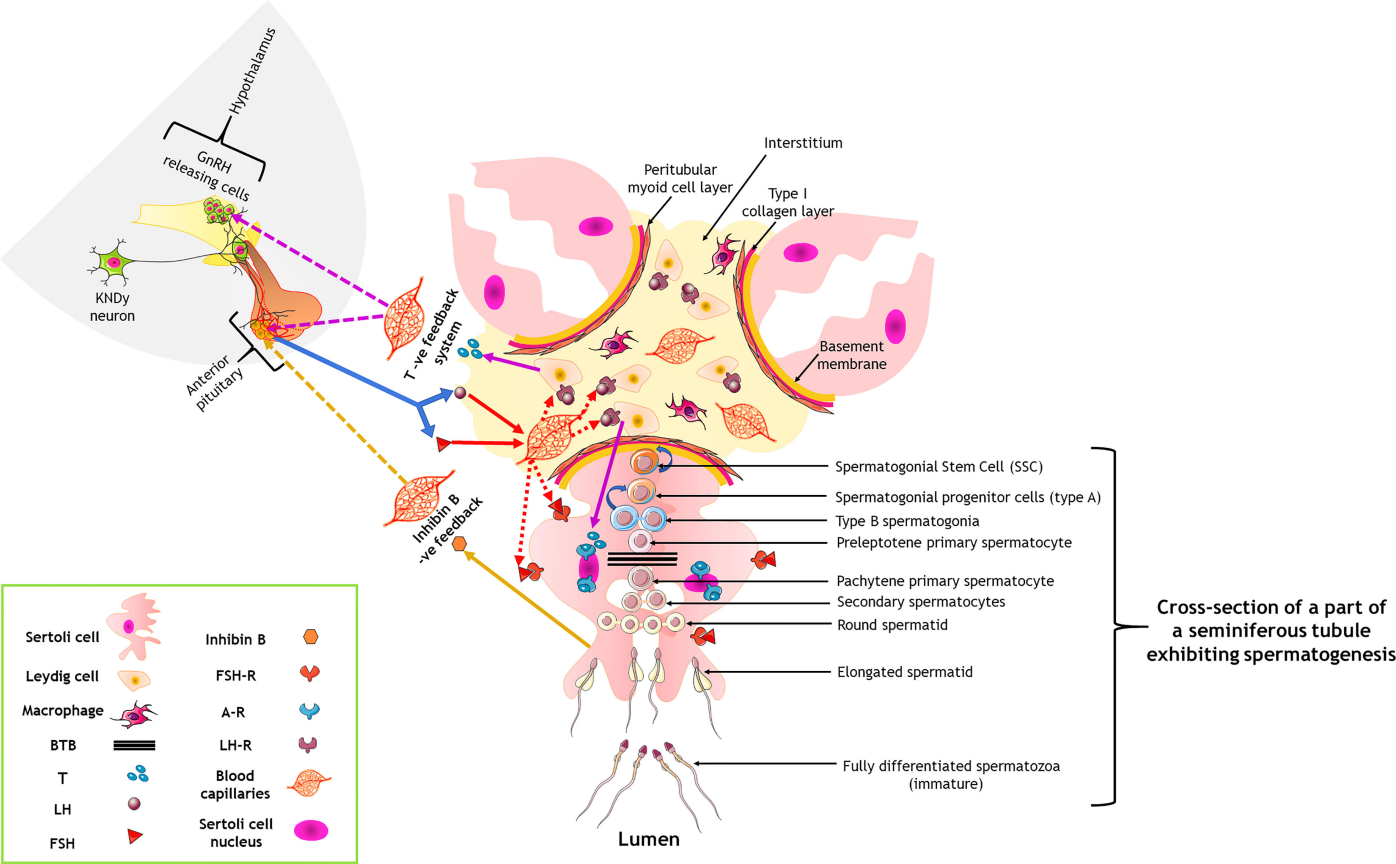
Hormonal control of spermatogenesis by the hypothalamo-hypophysial-testicular axis through a three-tier neuro-endocrine circuit. Curved blue arrows indicate a renewal of the cells; solid and dotted colored arrows denote the primary action and feedback action of the hormones. A-R, androgen receptor; BTB, blood-testis barrier; FSH, follicle stimulating hormone; FSH-R, FSH receptor; LH, luteinizing hormone; LH-R, LH receptor; T, testosterone. Only one seminiferous tubule has been shown to contain the germ cells; for others, it has been intentionally not shown, only to keep the figure less complicated for viewing of the readers.

**Figure 3:**
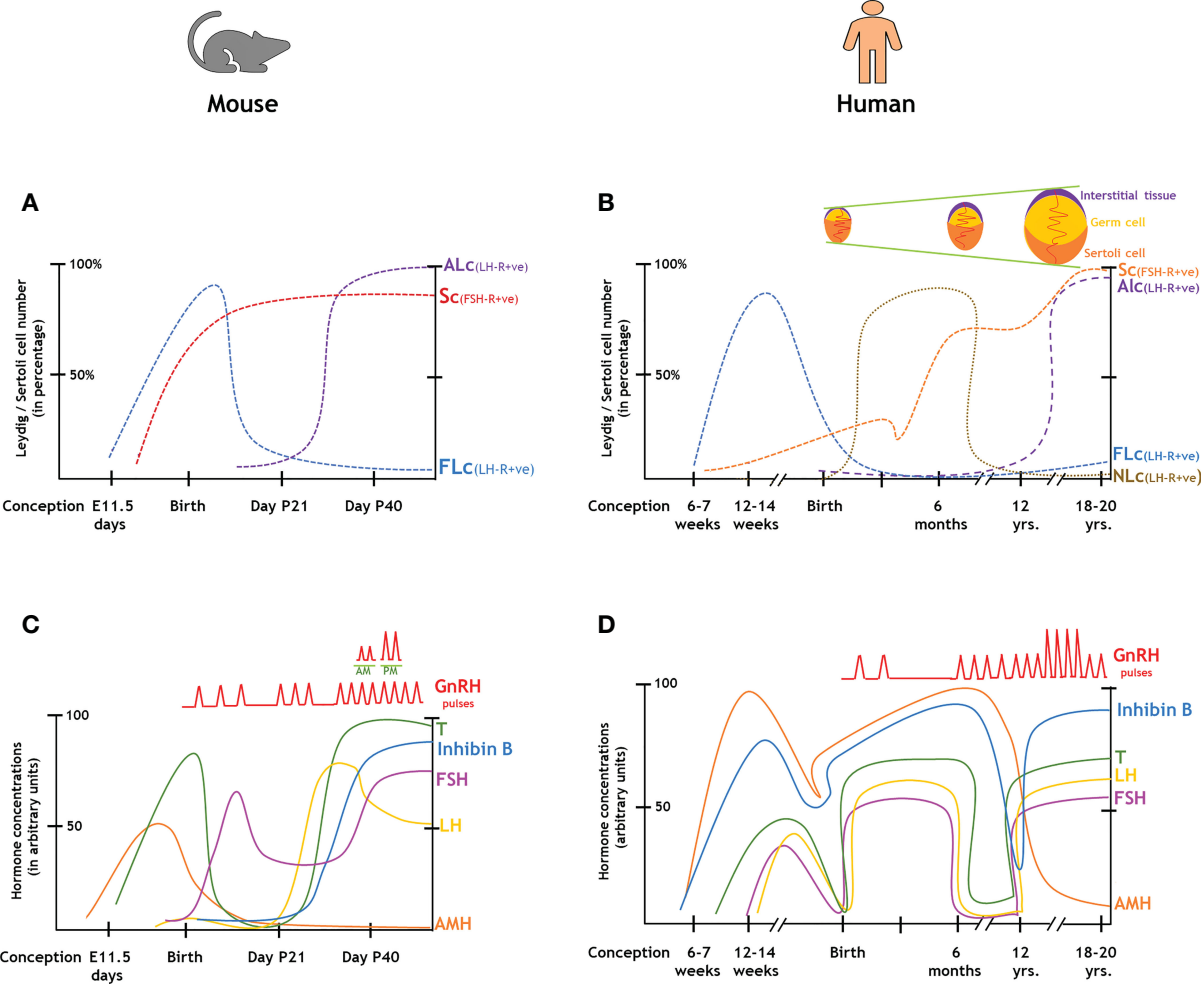
Changes in the endocrinal profiles in the course of the development of male gonads from the fetal stages to adulthood. **(A, B)**: Comparison of gonadal cell numbers in rodents and humans. **(C, D)**: Comparison of hormonal levels in rodents and humans. ALc, adult Leydig cell; AMH, anti-Mullerian hormone; FLc, fetal Leydig cell; FSH, follicle stimulating hormone; GnRH, gonadotropin-releasing hormone; LH, luteinizing hormone; NLc, neonatal Leydig cell; Sc, Sertoli cell; T, testosterone.

**Figure 4:**
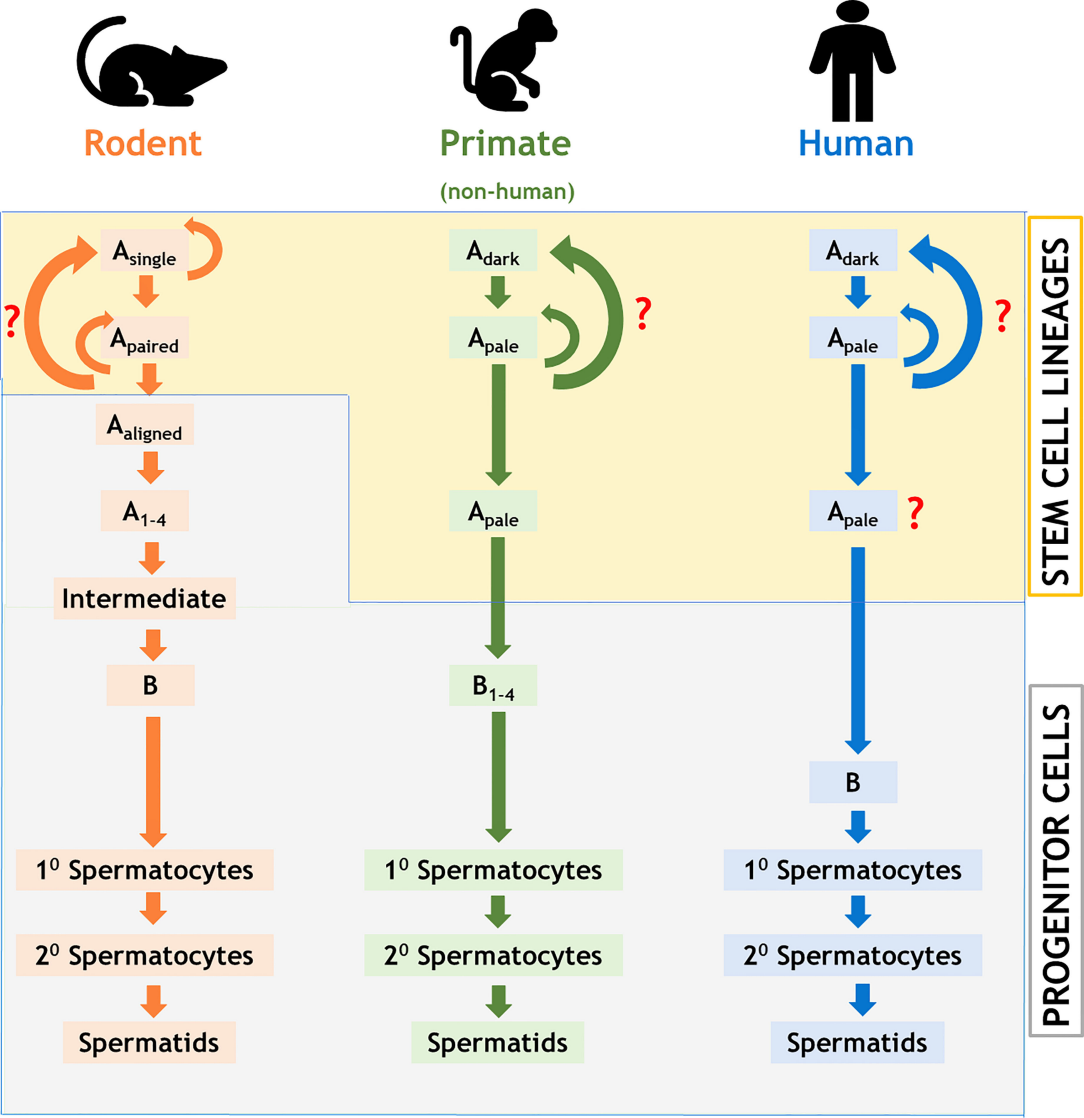
Comparison of stages of testicular development of the male germ cells among rodents, non-human primates, and humans. Note that the stem cell property differs between rodents and primates; the number of detectable stages of differentiation of the male germ cells varies significantly among all these three groups of animals. Colored curved arrows denote cell renewal; red question marks indicate unknown pathway.

We have prepared a PRISMA flow diagram (**Figure 1**) to systematically document the advancement of knowledge in the role of gonadotrophic hormones in the regulation of spermatogenesis in mammals. The flow chart is self-explanatory; in brief, we looked into the PubMed^®^ database for papers dealing with the topic in hand in the last decade. We only included original research papers, whose full text is deposited in the said database and concerns studies performed only on mammalian species. Thus, we narrowed down the total number of cited articles to 64 from 752 with the help of imposed inclusion and exclusion criteria. However, to address the regulation of mammalian spermatogenesis by gonadotropins from a broader developmental perspective and for the benefit of general readers, we have cited a substantial number of additional scientific articles in this review paper. **Figure 2** is the schematic representation of the HHT axis showing the site of sperm production. **Figure 3** represents the developmental (from the fetal stage to adulthood) changes in plasma hormonal profiles of mice and men. **Figure 4** displays a comparative picture of the initial critical steps in male germ cell differentiation in rodents, non-human primates, and humans.

## 2 FSH

### 2.1 FSH-receptor: Mode of signalling

FSH is a glycoprotein hormone having disulfide-rich heterodimers, a common α subunit (sharing with TSH and LH), and a unique β subunit. Evolving pieces of evidence suggest that pituitary-derived activins are the primary stimulators of FSH generation by gonadotrope cells. Activins control transcription of the FSH component gene (*Fshβ*) *in vitro via* SMAD3, SMAD4, and FOXL2 (22–25). FSH acts on Sc *via* FSH-R (**Figure 2**), a G protein-coupled receptor (GPCR), which transmits its signal by recruiting the intracellular GTP binding proteins (G-proteins, either stimulatory Gα_s_ or inhibitory Gα_i_) associated with it (26). Dual coupling of Gα_s_ or Gα_i_ to FSH-R differentially modulates the activity of adenylyl cyclase (AC) to regulate FSH-induced cAMP production within Sc (26). The concentration of cAMP subsequently directs the multiple downstream signaling cascades such as canonical Protein Kinase A (PKA) or other (PKC, PI3K, Akt/PKB, and ERK1/ERK2) pathways highlighting the pleiotropic effects of FSH in Sc (26). The robust cAMP response in Sc results in the activation of PKA which in turn phosphorylates cAMP Response Element Binding protein (CREB) to induce the transcription of genes such as *Stem cell factor* (*SCF*), *Glial cell line-derived neurotrophic factor* (*Gdnf*), *Androgen binding protein* (*Abp*), *Kruppel-like factor 4* (*Klf4*), *Transferrin* etc, that play a critical role in Gc differentiation (6, 26–30).

### 2.2 Developmental expression profile

In rats, FSH-R is first detected at E14.5 [embryonic age in days (E)], whereas the fetal plasma FSH concentration rises from E 19.5- 21, peaks at P5 [post-natal age in days (P)], then substantially drops during P15-20, finally recovered to a steady state by P40-50 (31, 32); similar events occur in mice (**Figures 3A, C** **)**. On the other hand, FSH is uniformly detectable in human fetal circulation from 12-18 week of gestation (WG), peaks during 20-22 WG and then gradually declines in term pregnancy **(** **Figures 3B, D** **)** (33, 34), whereas specific binding of FSH is observed in human and rhesus monkey (*Macaca mulata)* testes during 8–16 and 19–22 WG, respectively (35, 36). In post-natal life, FSH concentration first raises upto the adult range within a week of parturition and stays stable till 4-6 months, then declines and gets undetectable during the juvenile period prior to its re-elevation at puberty (4, 5). Although circulatory FSH levels remain relatively constant in adult men and rats (4, 5), the expression pattern of FSH-R cyclically changes in a stage-specific manner, maximal during stages XIII–II and minimal at VII–VIII (37). FSH has been shown to suppress FSH-R transcription at 6-8 hr (38) in cultured Sc and subsequently gets recovered by FSH at 24-48 hr (39).

### 2.3 Mode of function

*In utero* life, FSH has been shown to induce Sc proliferation and augments AMH (Anti Müllerian Hormone) production in both rodents (40) and primates (41) and this fetal expansion of the Sc population critically regulates the maximal spermatogenic output in adult testes (42–45). Such FSH-driven Sc proliferation gets continued in neonatal (upto P15) rats and infant primates (upto 3-6 months) and ceases with functional maturation of Sc during pubertal development (27–30). It is interesting to note here that unlike puberty, FSH induced cAMP production is limited during infancy in both rats (27, 28) and rhesus monkeys (29, 30) and therefore Sc fails to support robust Gc differentiation at younger ages despite being exposed to sufficiently high levels of FSH and FSH-R (27–29). Unlike pubertal cells, diminished plasma membrane localization of FSH-R protein in rats (27) and limited expression of Gαs protein in monkeys are considered to be the underlie causes of such poor cAMP response by FSH in infant Sc (29).

### 2.4 Action in rodents

In hypophysectomised or GnRH depleted (via pharmacological or immunological inhibition) rats, administrations of FSH alone show partial spermatogenic restoration (46, 47). For example, FSH replacement in GnRH antagonist-treated rats significantly rescues spermatogonia B and early spermatocytes (48). Immuno-neutralization of FSH in post-natal rats indicates FSH promotes Sc proliferation and Gc survival in neonatal age, whereas pre-meiotic Gc differentiation in pubertal age (49). Exogenous administration of FSH alone in pre-pubertal *hpg* mice fails to induce sperm production (50). Similarly, pituitary independent transgenic expression of human (h) FSH (51) or mutated [at Asp567Gly and constitutively active (capable of FSH independent cAMP production)] h-FSH-R (h-FSH-R*) (52) in male *hpg* mouse leads to incomplete meiotic progression. Furthermore, although h-FSH-R* over-expression augments proliferation/development of Sc/pre or early meiotic Gc in wild-type testes (53) this hyper-active receptor fails to maintain normal spermatogenesis during experimental deprivation of gonadotropins (54). However, over-expression of h-FSH-R* shows LH-independent steroidogenic activity (55). Notably, over-expression of FSH-Rs [either h-FSH-R* (along with normal *h-FSH-R*) or another hyper-mutated (at Asp-580-His, constitutively active (capable of FSH independent cAMP productive) mouse (m) FSH-R (m-FSH-R*)] do not affect normal spermatogenic maintenance (55). Finally, both FSH or FSH-R Knock-out (KO) mice demonstrate reduced testis size with reduced numbers of Sc and Gc (spermatogonia, spermatocytes and round spermatids) leading to sub-fertility (56–58) concluding dispensable role of FSH in rodents. However, this dogma has recently been challenged as the expression of hyper-active m-FSH-R* shown to rescue male fertility in LH-Receptor (LH-R) KO mice with a complete absence of testicular androgens (due to exogenous flutamide treatment) (59).

### 2.5 Action in primates

FSH has been shown to be mitogenic for Sc and induce early differentiation in spermatogonia A in rhesus and cynomolgus monkeys (long-tailed macaque; *Macaca fascicularis*) (15–17). However, five finish men with an inactivating mutation in FSH-R have been reported to have variable degrees of spermatogenic failure without complete loss of fertility (60). In multiple hypogonadotropic hypogonadal clinical studies (61–64) and/or experimentally induced and/or gonadotropin deficient non-human primates (65–68), supplementations of FSH alone (independent of LH/T) results to limited spermatogenic recovery without appearance of either elongated spermatid or spermatozoa. FSH has been shown to regulate the number of pachytene spermatocytes in adult men (69). These reports suggest that like rodents, FSH plays only a supportive role in regulating male fertility in men. However, there are substantial contradictory reports available in men indicating an absolute requirement of FSH for sperm production. For example, hCG-mediated suppression of circulatory FSH in adult men results into poor sperm counts, with one individual developing complete azoospermia, which later gets recovered by FSH supplementation alone (70). Similarly, a hypophysectomized man with complete gonadotropin deficiency fathered three children having h-FSH-R* (71). Finally, complete infertility has been observed in men lacking normal circulating FSH due to mutated *FSH-β* (72–74). Furthermore, two cases of isolated FSH deficiency with normal *FSH-β* gene and usual LH/T levels [first, two young men having moderate testicular hypotrophy (75, 76), second, a 19 years old boy being homozygous for a novel silent polymorphism (G/T substitution) in *FSH-β* promoter (77),] show severe sperm abnormalities to complete azoospermia respectively. Intriguingly, immuno-neutralization of circulatory FSH shows acute spermatogenic abnormalities in both bonnet monkeys (*Macaca radiata*) (78) and men (79) suggesting FSH vaccination as a promising male contraceptive strategy (80). Taken together, the critical contribution of FSH in regulating primate spermatogenesis is still currently disputed (15, 17, 81, 82).

## 3 LH

### 3.1 Developmental expression profile

LH binds to LH-R expressed by interstitial Lc and indirectly exerts its actions on spermatogenesis through T–AR interaction *via* regulating Sc functions **(** **Figure 2** **)** (6, 82). In rats, fetal plasma LH concentration gets elevated from E 18- 21, then rises at P5-7, further gets reduced during P 20-25, rises again by P35 to peak at P60 and remains constant thereafter throughout adulthood prior to aging (P 400-500) (31, 32). In humans, pituitary LH is measurable from 12-18 WG (which is around 10-fold lower than placental hCG), peaks during 20-22 WG and then gradually decline in term pregnancy (**Figures 3B, D** **)** (33, 34). However, such a pattern remains inconsistent with the corresponding T profile which peaks during 12-14 WG and then drops during the second trimester corroborating with placental hCG (83). In post-natal life, LH concentration first raises upto the adult range within a week of parturition and then stays stable till 4-6 months, subsequently gets undetectable during the juvenile period, and finally shows the pubertal elevation by reaching its maximal range (4, 5).

### 3.2 Target cells

Classical histological studies have identified two developmentally diverse populations of Lc e.g.- fetal (FLc) and adult (ALc) (83). FLc originate from coelomic epithelium and notch active Nestin-positive perivascular cells located at the gonad–mesonephros borders, and get specified as Nr5a1 or Ad4BP/SF-1 expressing cells by E 12.5 in fetal mouse testes (84). These cells produce androstenedione (precursor of T, due to lack of HSD17β3 enzyme) and play a critical role in initial virilization and patterning of the male external genitalia (84). However, in neonatal (P 5-15) testis, FLc undergo massive dedifferentiation and during puberty (P 15-21) gradually get replaced by T producing ALc (85, 86). FLc also secretes INSL3, a member of the insulin-relaxin family of peptides that acts on the body through the G-protein-coupled receptor relaxin/insulin-like family peptide receptor 2 (RXFP2). Missense mutations or ablation of *Insl3* or *Rxfp2* causes cryptorchidism leading to azoospermia (87, 88). However, unlike rodents, primate Lc shows a triphasic developmental pattern (83–86). In human, FLc peak during 12-14 WG (83) and subsequently get dedifferentiated by the end of the second trimester and is replaced by a unique population of neonatal-Lc (NLc) just during/after birth which persist for first 4-6 months of infantile age, when the HHT axis remains active (89). During the onset of juvenile period (inactivation of the HHT axis) massive involution occurs in the NLc population and finally ALc population originates from the dedifferentiating NLc population during puberty (83).

### 3.3 Signalling and critical function

Like FSH-R, LH-R/LHCG-R is also a GPCR that recruits cAMP-dependent PKA pathway to induce the expression and activation of steroidogenic acute regulatory protein (STAR) at the outer mitochondrial membrane of ALc leading to cholesterol trafficking for initiation of steroidogenesis and eventually biosynthesize T (90). However, despite being responsive towards LH signal, FLc of both rodents and primates are independent of fetal LH action (83). FLc number or external genitalia remain unaffected in hpg (13), LH-RKO (91), LH-βKO (92) and ARKO (93, 94) adult male mice suggesting murine FLc are functionally independent of LH or T. In contrast, although patients having *LH-β* mutations show normal masculinized development (95–99), *LHCG-R* mutations lead to pseudo-hermaphroditism (100) indicating definite role of hCG on FLc functioning in men. However, in both the species LH is absolutely required for ALc function (83) as evident from various mouse models [hpg (13), LH-RKO (91), LH-βKO (92) and ARKO (93, 94)], etc and mutations in human *LH-β*/*LHCGR* genes resulting masculinized fetus but compromised pubertal development and complete azoospermia due to total absence of functional pituitary LH and testicular T (100). It is interesting to note here that fertility can be restored in men with isolated LH deficiency due to mutations in the *LHβ* gene by long-term hCG supplementations within the critical “window of testicular susceptibility” during pubertal development (101).

Stimulation of LH (resulting T) in rhesus and cynomolgus monkeys leads to spermatogonial differentiation and initiation of Gc meiosis without insignificant rise in Sc number (15, 17, 102–105). LH/hCG (or T) mediated absolute recovery of spermatogenesis has been demonstrated in gonadotropin withdrawal models (either by hypophysectomy or treatment of GnRH receptor antagonist or active immunization against GnRH) in adult rodents (106–111), men (64, 112, 113) and non-human primates (114–118). Exogenous supplementations of T or LH/hCG alone have been shown to induce complete spermatogenesis in immature *hpg* mice (119, 120) or natural or induced hypogonadal men (121, 122). Genetic ablations of *LH-β* or *LH-R* in mice further show cryptorchid testes with spermatogenic arrest and male infertility (91, 92). Human patients having inactivated *LHCG-R* or *LH-β* frequently show pseudohermaphroditism and cryptorchidism with Lc hypoplasia and spermatogenic arrest (123–132). Interestingly, a unique homozygous deletion on exon 10 in *LHCG-R* has been reported in an azoospermic man having normal phenotype with diminished LH signaling (but not towards hCG) indicating higher potency of hCG on ALc (123). In contrast, activating mutations in *LH-β* or *LHCG-R* were shown to be associated with precocious puberty and Lc hyperplasia (133–148). Such precocious puberty with Lc hyperplasia followed by infertility has been observed in mice over-expressing hyper-active (Asp582Gly) LH-R (149). However, spermatogenesis has been reported in a man with a splice-mutation (homozygous point mutation G to A at -1 position of intron-10 to exon-11 junction) in *LHCG-R* with severe loss of T production (150). A more surprising study has been reported in a 43 years old man with a homozygous deletion of nine bases in *LHβ* gene generating a deletion of amino acids from 10 to 12 (*His, Pro, Ile*) in the amino-terminal critical for conformational changes leading to undetectable LH (high FSH) with very low T (151). Paradoxically, this isolated LH deficiency case eventually shows sub-optimal but spontaneous spermatogenesis (151). It is important here to note that, despite high (20-100 fold) intra-testicular T (IIT) concentration has been considered to be critical for spermatogenic initiation (152, 153), low levels of T are sufficient to drive spermatogenic maintenance as evident by spontaneous spermatogenesis in LH-RKO mice at 12 months of age (154).

### 3.4 Mode of T action

LH operates spermatogenic regulations through testicular androgen T and AR (155). T is essential for suppression of AMH (156, 157), pubertal maturation of testicular somatic cells (e.g.- PTc, Sc, Lc in developmental order) (2), the establishment of Blood-testis barrier (BTB) (158), meiotic progression of Gc and spermiation (159). The free titer of T depends upon the extent of the presence of sex hormone-binding globulin (SHBG) which binds to T with strong affinity; thus, SBHG regulates the process of spermatogenesis by controlling the serum concentration of biologically active T (160, 161). The absolute requirement of T on male fertility has been confirmed from ARKO (ubiquitously lacking AR) mice (93, 94). Despite most of the somatic testicular cells (Sc, PTc, Lc etc) express AR, Gc do not have functional AR (2, 3). Cell-specific selective ablation of AR [Sc specific i.e. SCARKO (162–164), Lc specific i.e. LcARKO (165, 166), PTc specific i.e. PTARKO (167, 168) or Gc specific i.e. GcARKO (169, 170)] demonstrated that AR expressed by Sc plays a pivotal role in the progression of Gc meiosis (20, 21, 155). Furthermore, the crossing of hpg mice with ARKO or SCARKO mice followed by T/5α- dihydrotestosterone (DHT) supplementation confirmed the critical significance of Sc-mediated AR signaling in spermatogenesis (171). The transition of round to elongated spermatid is fully dependent on T action transmitted *via* Sc (159).

In Sc, AR signals *via* both classical and non-classical manner (155). In the classical pathway, T (or 5α-DHT) activated AR binds to specific DNA sequences having *Androgen Response Elements* (*ARE*) and initiates the androgen-dependent transcriptional events e.g. *Rhox5* expression (155). However, in a non-classical pathway, T gets coupled with membrane-bound AR and triggers the binding of the proline-rich region of AR with the SH_3_ domain of membrane bound SRC kinase leading to stimulation of EGF receptor and subsequently activates MAP (RAF, MEK, ERK) kinase or CREB cascade inducing several genes which lack typical *ARE*s on their promoters e.g. *Ldha*, *Claudin11*, etc (155). *In vitro* studies show that T regulates spermiation *via* a non-classical pathway (155), however, *in vivo* studies suggest that classical pathway is most crucial for meiotic completion of Gc and fertility (159).

## 4 Synergy between FSH and LH/T

A productive synergy between FSH and LH (via T) has been observed in regulating maximal spermatogenic output (6, 14, 16, 17). For example, combined FSH and LH/hCG/T stimulations show better spermatogenic restoration than independent hormonal treatment in induced GnRH-depleted adult rats (16, 111) or primates (172–174). Patients suffering from *hypogonadotropic hypogonadism* show appreciable testicular maturation with sufficient Gc differentiation with combined FSH and hCG administrations (175–177). Pulsatile stimulations of LH and FSH together for only 11 days demonstrate enhanced Gc differentiation (upto spermatogonia B and primary spermatocytes) as compared to independent treatment of either LH or FSH in juvenile male monkeys (104). Moreover, T augments genes involved in FSH signalling pathway (e.g.- *FSH-R*, *Gαs* and *Ric8b etc)* resulting in elevated cAMP response in pubertal monkey Sc (178). These reports suggest that a coordinated network of FSH and T signalling in Sc facilitate the timely onset of the first spermatogenic wave in pubertal primates (14, 16, 17). Finally, spermatogenesis in Sc specific isolated or double (both *FSH-R* and *AR*) knockout mice gets affected more severely than single genetic ablation (either FSH-R or ARKO/SCARKO) confirming a dynamic synchronization between FSH and T action regulating the spermatogenic output thus male fertility (179–181)

## 5 Conclusion and future directions

For the past 50 years, various laboratories across the globe have significantly contributed in revealing the gonadotropic regulation of spermatogenesis (16, 17) with potential clinical implications (182, 183). **Table 1** describes the critical role(s) of FSH and LH (T) in spermatogenesis, whereas **Table 2** highlights the significant discoveries/advancements accomplished during past five decades in a chronological order.

**Table 1:** Critical roles of FSH and LH in the regulation of mammalian spermatogenesis.

Name	Gene and Protein	Receptor	Target Cells	Major Functions
FSH	Common α Specific β	FSH-R	Testicular Sertoli cells (Sc), Bone, and Epididymis.	i) Fetal and pre-pubertal expansion of Sc population to set the upper limit of sperm production. ii) Augmenting expression of SCF, GDNF, BMP4, Cyp19 Aromatase, FGF2 etc in Sc to regulate the induction of the proliferation/differentiation of undifferentiated spermatogonial cells. iii) Survival signal for proliferating pre- meiotic Gc. iv) Proliferation of Epididymal cells.
LH (via T)	Common α Specific β	LH-R	Testicular Leydig cells (Lc)	i) Production of testicular androgen, T. ii) Induction of virilization of male genital tract from embryonic Wolffian duct. iii) Driving suppression of AMH in pubertal Sc. iv) Promoting functional maturation of Sc during pubertal development. v) Establishment of BTB. vi) Meiotic progression of developing Gc, transforming round spermatid to elongated spermatid. vii) Regulating spermiogenesis and spermiation. viii) Controlling male sex drive/libido.

Note that various target cells of each of these hormones are affected differentially by it.

**Table 2:** Chronological representation of the pioneering progress in gonadotropin biology during past decades.

Duration/ Decade	Main Model used	Aim and Experimental setup	Significant Outcome	Key Review References
1920-1950s	Equine/Ovine/Porcine/Rodents species and human patients/clinical case studies	Isolation/Characterization of gonadotropins	Identifications of FSH/PMSG/LH/hCG etc	(7)
1960s	Ovine/Porcine/Rodents, species and human patients/clinical case studies.	Isolation/Characterization of LHRH (GnRH) and gonadotropins	i) Purification of GnRH, ii) Establishment of RIA to measure serum hormonal profiles	(7, 12)
1970s	i) Rodents/Non-human primates/Human, ii) Hypogonadal boys or men/clinical male patients	i) Withdrawal effects of FSH and LH after hypophysectomy, or GnRH antagonist treatment, GnRH immuno- neutralization ii) Initiation of spermatogenesis by FSH/LH (purified) in clinical hypogonagal boys/men.	i) Serum hormonal profiling from fetal stage to adulthood ii) Effect of hormones in testicular function and Gc development ii) Discovery of natural mutations like hpg and tfm mice	( 4, 5, 13–17, 89, 182, 183)
1980s-mid 1990s	i) Rodents/Non-human primates/Human, ii) Hypogonadal boys or men/clinical male patients	i) Withdrawal effects of FSH and LH after hypophysectomy, or GnRH antagonist treatment, GnRH immune-neutralization, FSH immunoneutralization/vaccination, T mediated suppression of GnRH . ii) Restoration of spermatogenesis after GnRH/FSH/T withdrawal by exogenous supplementations of FSH/LH/hCG (purified/recombinant) either alone or in combination iii) Initiation of spermatogenesis by FSH/LH/hCG (purified/recombinant) in hpg mouse or clinical hypogonadal men iv) Pulsatile stimulation of GnRH in male juvenile monkeys for induction of synchronized precocious puberty v) Culturing Sc and Lc for evaluating FSH/T and LH induced downstream signalling events/gene transcriptions	i) Independent and/or synergistic effects of hormones in testicular function and Gc development ii) FSH essential for maintaining Sc & pre-meiotic Gc numbers iii) LH/hCG (via T) critical for complete recovery of male fertility iv) productive synergy between FSH and T in optimizing spermatogenic output v) Identifications of inactivating or hyper-active mutations in FSH-R/LHCG-R genes in human/mouse. vi) FSH-R, LH-R and AR-mediated signalling cascades in Sc and Lc	(6, 14–17, 89, 131, 182, 183)
Mid 1990s- 2020	i) Rodents/Non-human primates, Human ii) Hypogonadal boys or men/clinical male patients iii) Boys and men with either inactivating or hyper-active mutations in either FSH-R or LHCG genes	i) Pusatile stimulation of GnRH or FSH/LH in male juvenile/adult monkeys for induction of synchronized precocious puberty or Gc differentiation ii) Culturing Sc and Lc and evaluating FSH/T and LH induced downstream signalling events/gene transcription iii) Whole or cell type-specific knockout mice models of FSH-β. LH-β, FSH-R, LH-R, AR, etc. iv) Investigating FSH or LH/T inducible/responsive genes in Sc/Lc culture or in knockout mice models for FSH-R/AR etc by Microarray/RNA-seq analyses v) Single-cell transcriptomics in different testicular cells	i) Independent and/or synergistic effects of FSH and LH (T) in testicular function and Gc development ii) Identification of FSH and T responsive genes in Sc and Gc development iii) Redundancy of FSH in rodent spermatogenic progression/completion/spermiogenesis iv) Critical role of FSH in human spermatogenesis v) Absolute requirement of T in Gc meiosis via Sc vi) Identifications of inactivating or hyper-active mutations in FSH-R/LHCG-R genes in human/mouse. vii) Genomic and Non-genomic mode of actions of T in Sc critical for male fertility viii) Cell type specific unique transcriptional profiling in different stages differentiating Gc, ix) Differential gene expression during phases of Sc and Lc maturation x) Discoveries of hormone-responsive novel putative noncoding RNAs regulating male fertility or infertility	(6, 17–21, 26–30, 80, 81, 83, 85, 86, 89, 131, 159–171, 181, 184–186)

In summary, hypothalamic KNDy neurons induce GnRH discharge which further stimulates the secretion of gonadotropins (FSH and LH) from pituitary. High and low pulse frequencies of GnRH selectively favor either LH or FSH release. Multiple experimental/natural models (e.g.- hypophysectomised or pharmacological/immunological deprivation of GnRH, hpg mice or hypogonadal men), inactivating or hyper-activating mutations in *FSH-R*/*LHCG-R* in men, murine genetic KOs collectively show the crucial role of FSH and LH (via T) in spermatogenic development and maintenance. In rodents, FSH essentially supports Sc proliferation and survival, division, and differentiation of pre-meiotic Gc, but fails independently to direct the completion of spermatogenesis. However, the sole role of FSH still remains controversial in men. On the other hand, LH (via T) founds to be indispensable for regulating male fertility in both species and Sc-mediated AR signaling found to be is most critical for the transition of round to elongated spermatids and the induction of spermiation. A productive synergy between FSH and T has been established to optimize the spermatogenic capacity both qualitatively and quantitatively. A recent report indicated the presence of a mesenchymal transcription factor (Tcf) 21 positive interstitial progenitor population acting as a potential reservoir during injury-induced ALc regeneration (187).

However, despite such extensive information generated during past decades translational progress in terms of clinical success has not been achieved yet in the field of gonadotropin biology toward treating infertility in men or developing reversal male contraceptives (1). This is largely due to limited numbers of hormone [FSH and LH (T)]-responsive genes identified so far with defining impact on spermatogenesis identified till date from multiple *in vitro* (184) and *in vivo* (185) studies. Future studies utilizing a cutting-edge single-cell transcriptomics approach are required to identify and investigate such putative gonadotropic inducible genes crucial for regulating male fertility with the following probable objectives/outcomes: significant advancement in classifying and curing idiopathic male infertility, bioengineering of fertilizable spermatozoa ex vivo, and sustainable development of potential male contraceptive targets (186, 188).

## Author contributions

IB conceived the idea and designed and prepared the initial draft. SD prepared the figures, revised the manuscript and generated the final form with inputs from AB. All authors contributed to the article and approved the submitted version.
